# Ultrasonography of the rumen of dairy cows

**DOI:** 10.1186/1746-6148-9-44

**Published:** 2013-03-05

**Authors:** Ueli Braun, Adrian Schweizer, Luzia Trösch

**Affiliations:** 1 Department of Farm Animals, Vetsuisse Faculty, University of Zurich, Winterthurerstrasse 260, Zurich CH-8057, Switzerland

## Abstract

**Background:**

This study describes the ultrasonographic findings of the rumen in 45 healthy dairy cows.

**Results:**

The cows were scanned on both sides using a 5.0 MHz transducer. The dorsal visible margin of the rumen ran parallel to the lung from cranioventral to caudodorsal. It was furthest from the dorsal midline at the 9th intercostal space (48.3 ± 9.24 cm) and closest at the 12th intercostal space (22.4 ± 3.27 cm). The longitudinal groove, which could be clearly identified at all examination sites because it appeared as a triangular notch, formed the ventral margin of the dorsal sac of the rumen. The dorsal sac of the rumen was largest at the caudal flank (40.3 ± 6.33 cm), where it was adjacent to the abdominal wall. The ventral sac of the rumen extended across the ventral midline into the right hemiabdomen and its ventral margin had a largely horizontal craniocaudal course. The height of the ventral sac of the rumen exceeded that of the dorsal sac at all examination sites; the maximum height was measured at the 12th intercostal space (62.6 ± 9.53 cm). The dorsal gas cap, characterised ultrasonographically by typical reverberation artifacts, was visible in all cows from the 12th intercostal space to the caudal flank. It was largest at the 12th intercostal space (20.5 ± 7.03 cm). The transition from the gas cap to the fibre mat was marked by the abrupt cessation of the reverberation artifacts. It was not possible to differentiate a fibre mat and a ventral fluid phase. The rumen could be imaged from the right side in 21 cows (47%).

**Conclusions:**

Ultrasonography is well suited for the detailed examination of the rumen of cows. The reference values obtained from this study add to the diagnostic tools that are available for the assessment of bovine patients.

## Background

The rumen of adult cattle occupies most of the left hemiabdomen and extends across the ventral midline into the right hemiabdomen caudoventrally [1]. The anterior dorsal blind sac, separated from the dorsal sac of the rumen by the anterior ruminal groove [2], is continuous with the reticulum, from which it receives recently ingested feed. Two similarly sized blind sacs of the dorsal and ventral sacs of the rumen form the posterior part of the organ. Grooves that are visible externally divide the rumen into different sacs; the left and right longitudinal grooves separate the ventral and dorsal sacs of the rumen. In healthy cattle, the ruminal contents are stratified into a ventral fluid layer, a thick fibre mat in the middle and a dorsal gas cap.

There are many diseases that affect the rumen directly or indirectly including ruminal tympany, frothy bloat, reticulo-omasal stenosis, ruminal acidosis and alkalosis, microbial inactivity, putrefaction and peritonitis [3]. Standard techniques used to examine the rumen and rumen function include auscultation, inspection and palpation via the left flank, palpation transrectally and the collection and analysis of rumen fluid [4]. Variables such as pH and chloride concentration of rumen juice can be quantified, whereas rumen size, severity of tympany, degree of liquefaction of the rumen contents in cattle with grain overload, or amount of ingesta impacting the rumen in cattle with reticulo-omasal stenosis can only be assessed subjectively. This underscores the need for additional diagnostic techniques to accurately determine the size of the rumen and the ingesta layers including gas cap, fibre mat and fluid phase. Radiography is not suitable for the examination of the entire rumen because of the size of the organ. Although computed tomography has been used successfully in goats and calves to accurately measure the rumen and the dimensions of the ingesta layers [5,6], this imaging technique is limited to the examination of the head and legs in mature cattle because of the disproportion between the size of the equipment and the size of the patient [7]. In contrast, ultrasonography has been used for ruminal examination in calves [8] as well as mature cows [9,10]. Recent studies were limited to the examination of ruminal stratification of the ingesta in three Swiss Braunvieh cows [9] and description of the ruminal wall and characterisation of ruminal motility in ten Indian Jersey-Red Sindhi cross cows [10]. Size and abdominal location of the rumen have been investigated in calves [8] and goats [11] but not in mature cattle. Therefore, the goal of this study was to establish ultrasonographic reference values for the rumen of dairy cows. To enhance representativeness of the reference values, cows of three common dairy breeds used in Switzerland were included in the sample studied.

## Methods

### Animals

Fifteen Swiss Braunvieh, 15 Simmental and 15 Holstein-Friesian cows were used. The cows were three to nine years of age, up to five months pregnant, clinically healthy and weighed between 550 and 750 kg (610 ± 46 kg). They were kept in tie stalls, bedded with straw and fed hay and grass silage supplemented with a concentrate according to the level of production. The cows were not fasted for the ultrasonographic examinations.

### Clinical examination

All cows underwent clinical examination including assessment of the demeanour and general attitude, rectal temperature, heart and respiratory rates, rumen motility and fill, stratification of the rumen contents and foreign body tests. The cows were examined transrectally and by simultaneous percussion and auscultation on both sides. A urine sample was examined for colour, transparency, specific gravity and other characteristics using a test strip, and a rumen fluid sample was examined for colour, odour, consistency, pH, methyl blue reduction time and chloride concentration. Because only healthy cows were used in this study, results of the examinations are not given but were presented elsewhere [12].

### Ultrasonographic examination

The cows were clipped on both sides and ventrally. Before each examination, the skin was cleaned with alcohol, and lubricant (Vetogel®, Streuli Pharma AG, Uznach) was applied to the skin. A real-time B-mode ultrasound machine (EUB 8500, Hitachi Medical Systems, Zug) and a convex 5.0-MHz transducer were used to scan the standing non-sedated cows. The rumen was examined on the left side from the 8th intercostal space to the flank and on the right side from the 10th intercostal space to the flank. The visibility of the rumen at each intercostal space was determined, and neighbouring organs were identified on both sides. The dorsal and ventral visible margins of the rumen were determined on the left side by measuring the distance from each margin to the dorsal midline, and the height of the rumen was calculated by subtracting the distance between the dorsal margin and the dorsal midline from the distance between the ventral margin and the dorsal midline, analogous to the technique previously described in calves [8] and goats [13]. The location of the longitudinal groove was also determined by measuring its distance from the dorsal midline. The height of the dorsal sac of the rumen was defined as the distance from the dorsal visible margin of the rumen to the longitudinal groove, and the ventral sac was defined as the distance from the longitudinal groove to the ventral visible margin of the rumen. The distance of the rumen from the abdominal wall was measured at the dorsal and ventral visible margins using the electronic cursors on the frozen images.

The stratification of the ingesta was assessed on the left at the 12th intercostal space and the flank. The rumen was examined from dorsal to ventral with the intention of identifying transitions from the gas cap to the fibre mat and from the fibre mat to the fluid layer. The thickness of each layer was calculated by subtracting the distance between the dorsal margin of the layer and the dorsal midline from the corresponding distance of the ventral margin of the layer.

### Statistical analysis

Means, standard deviations and frequency distributions of variables were calculated using Windows Excel 2010, and ANOVA and t tests were done to analyse differences using OpenEpi (Open Source Epidemiologic Statistics for Public Health, free and open source software).

## Results

There were no statistically significant differences between the three breeds of cows and therefore the results were pooled.

### Anterior dorsal blind sac of the rumen

**Figure 1:**
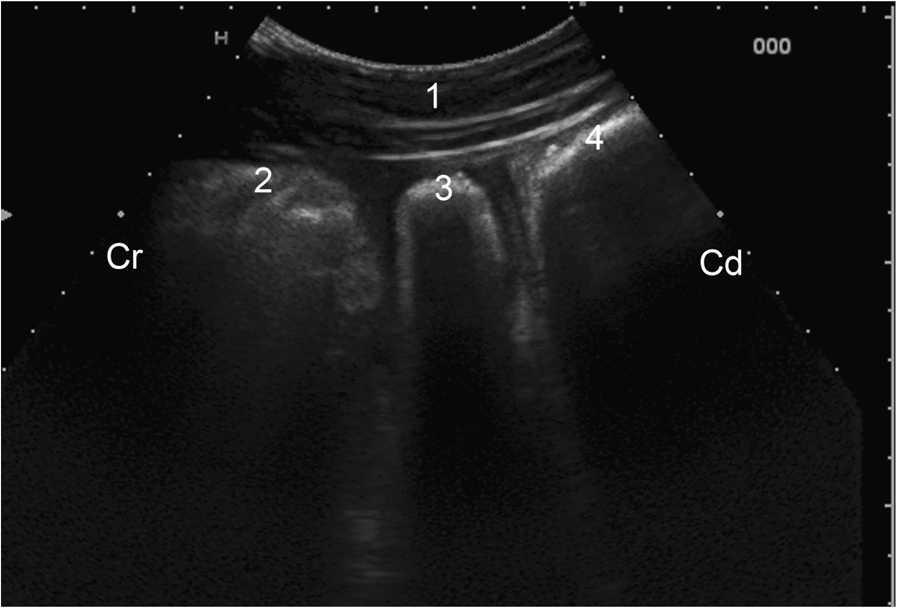
**Ultrasonogram of the anterior dorsal blind sac. **Ultrasonogram of the anterior dorsal blind sac of the rumen of a six-year-old Simmental cow viewed from the sternal region using a 5.0-MHz convex transducer. 1 Ventral abdominal wall, 2 Reticulum, 3 Anterior dorsal blind sac of the rumen, 3 Anterior ventral blind sac of the rumen, Cr Cranial, Cd Caudal.

The anterior dorsal blind sac of the rumen was visible between the reticulum and the anterior ventral blind sac of the rumen on the left side in all cows and was always adjacent to the abdominal wall (Figure 1). It was imaged in 22 cows (49%) at the 9th intercostal space and in 23 cows (51%) at the 10th intercostal space. The hyperechoic wall of the anterior blind sac of the rumen did not differ from the wall of the ventral sac of the rumen.

### Dorsal sac of the rumen scanned from the left

**Figure 2:**
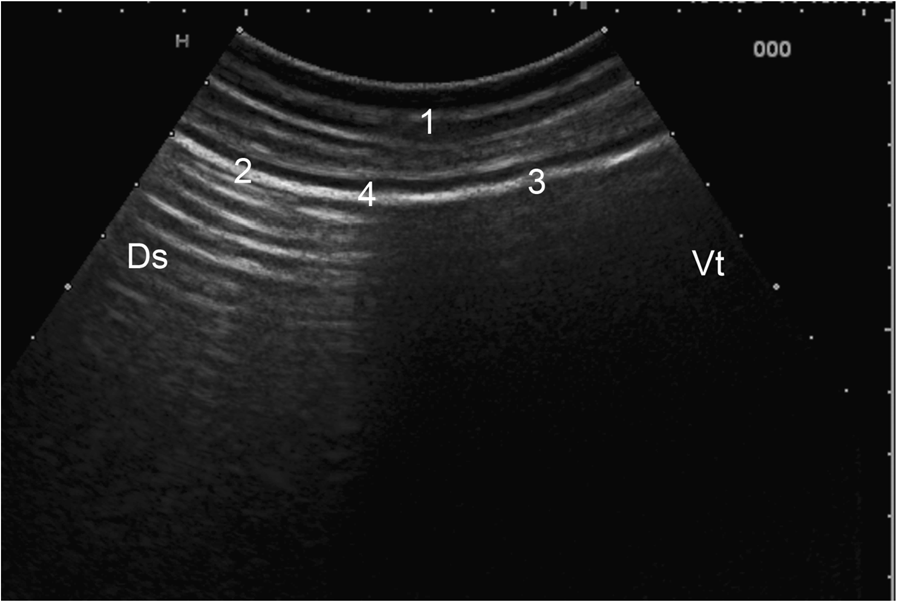
**Ultrasonogram of the rumen. **Ultrasonogram of the rumen showing the transition between the dorsal gas cap and ingesta layer viewed from the 12th intercostal space on the left side of a nine-year-old Swiss Braunvieh cow. The rumen wall appears as a hyperechoic line. Reverberation artifacts, which run parallel to the ruminal wall, reflect the gas cap and end abruptly at the transition to the fibre mat. 1 Abdominal wall, 2 Rumen wall at the level of the dorsal gas cap, 3 Rumen wall at the level of the fibre mat, 4 Transition between 2 and 3, Ds Dorsal, Vt Ventral.

**Figure 3:**
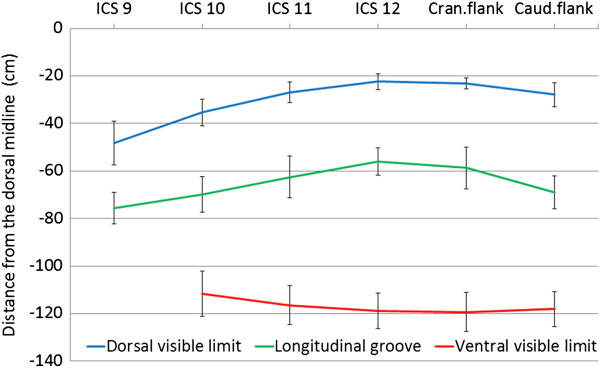
**Visible margins of the rumen. **Dorsal and ventral visible margins of the rumen and location of the left longitudinal groove relative to the dorsal midline imaged ultrasonographically from the 9th intercostal space to the caudal flank on the left in 45 cows (mean ± standard deviation).

The dorsal sac of the rumen was visible in all cows at the 10th to 12th intercostal spaces and the flank as well as at the 9th intercostal space in 21 cows (47%). It was adjacent to the left abdominal wall except for the craniodorsal region, where it was displaced medially by the spleen. The wall appeared hyperechoic, and the dorsal gas cap could be identified because of typical reverberation artifacts (Figure 2). Because of superimposition of the lungs, the course of the dorsal visible margin of the dorsal sac coincided with the ventral border of the lung and ran from cranioventral to caudodorsal (Figure 3, Table 1). The distance between the dorsal midline and the dorsal visible margin was largest (48.3 ± 9.24 cm) at the 9th intercostal space, decreased gradually caudally reaching a minimum (22.4 ± 3.27 cm) at the 12th intercostal space (Difference to ICS 9 P < 0.01) and then increased slightly toward the caudal flank (27.8 ± 5.01 cm). The left longitudinal ruminal groove formed the ventral margin of the dorsal sac of the rumen and its course was parallel to the dorsal visible margin (Figure 3). It was furthest from the dorsal midline at the 9th intercostal space (75.6 ± 6.52 cm), became gradually closer to the dorsal midline toward the 12th intercostal space (56.0 ± 5.88 cm) (Difference to ICS 9 P < 0.01) and then deviated ventrally in the flank (69.0 ± 6.84 cm).

**Table 1:** Results of ultrasonographic examination of the rumen in 45 cows (mean ± sd, range, all measurements in cm)

		**Intercostal space**				**Flank**	
**Part of rumen**	**Variable**	**9**^**a**^	**10**^**b**^	**11**	**12**	**Cranial**	**Caudal**
Dorsal sac	Dorsal margin	48.3 ± 9.24	35.4 ± 5.72	27 ± 4.30	22.4 ± 3.27	23.1 ± 2.31	27.8 ± 5.01
		(34 – 75)	(25 – 48)	(17 – 40)	(14 – 31)	(18 – 28)	(23 – 42)
	Ventral margin	75.6 ± 6.52	69.8 ± 7.40	62.5 ± 8.70	56.0 ± 5.88	58.7 ± 8.69	69.0 ± 6.84
		(64 – 86)	(47 – 93)	(23 – 76)	(36 – 66)	(46 – 87)	(46 – 85)
	Height	27.9 ± 8.74	34.5 ± 7.26	37.4 ± 6.10	33.4 ± 5.99	34.9 ± 7.49	40.3 ± 6.33
		(15 – 45)	(6 – 53)	(20 – 50)	(12 – 48)	(22 – 59)	(19 – 57)
	Thickness of wall	NE	NE	0.3 ± 0.06	0.3 ± 0.07	0.3 ± 0.06	0.3 ± 0.08
				(0.2 – 0.5)	(0.1 – 0.4)	(0.1 – 0.4)	(0.2 – 0.6)
	Distance to abdominal wall	10.3 ± 1.66	9.9 ± 2.60	8.8 ± 3.59	4.4 ± 2.74	1.1 ± 1.38	2.0 ± 2.12
		(6.7 – 14.2)	(0 – 15.4)	(1.8 – 15.1)	(0 – 13.8)	(0 – 4.5)	(0 – 10.8)
	Height of dorsal gas cap	NE	NE	NE	20.5 ± 7.03	19.2 ± 6.33	15.6 ± 5.35
					(7 – 40)	(4 – 34)	(5 – 25)
Ventral sac	Ventral margin	NA	111.7 ± 9.54	116.4 ± 8.10	118.8 ± 7.46	119.3 ± 8.24	118.1 ± 7.33
			(91 – 128)	(96 – 140)	(105 – 139)	(108 – 135)	(103 – 134)
	Height	NA	46.0 ± 8.65	52.6 ± 6.78	62.6 ± 9.53	61.4 ± 11.19	49.9 ± 5.38
			(24 – 61)	(38 – 73)	(50 – 95)	(39 – 95)	(36 – 64)
	Thickness of wall	NA	NE	0.4 ± 0.07	0.3 ± 0.06	0.3 ± 0.07	0.3 ± 0.08
				(0.2 – 0.6)	(0.2 – 0.5)	(0.3 – 0.5)	(0.3 – 0.5)
	Distance to abdominal wall	NA	4.6 ± 1.74	4.8 ± 1.79	5.7 ± 2.43	6.1 ± 2.49	5.7 ± 2.52
			(0 – 7.4)	(2.4 – 8.7)	(1.5 – 14.1)	(2.4 – 12.3)	(0 – 14.6)
Dorsal and ventral sac combined	Height	27.9 ± 8.74^c^	77.2 ± 9.90	90.2 ± 8.68	96.0 ± 8.01	97 ± 8.88	90.2 ± 7.97
		(15 – 45)	(56 – 96)	(71 – 111)	(84 – 120)	(77 – 114)	(77 – 108)

^a ^Dorsal sac of rumen imaged in 21 cows.

^b ^Ventral sac of rumen imaged in 22 cows.

^c ^Measurement relates to dorsal sac of the rumen because ventral sac was not imaged.

*NE*, Not examined.

*NA*, Not applicable.

**Figure 4:**
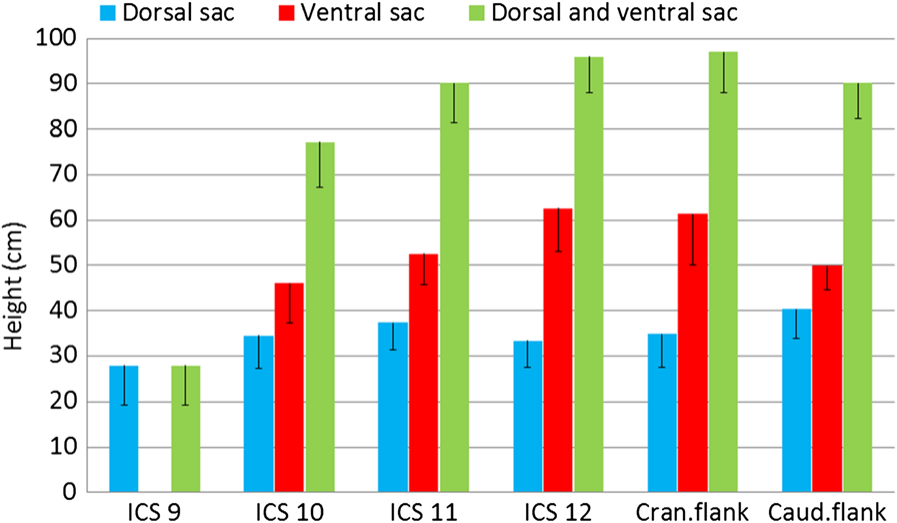
**Height of the rumen sacs. **Height of the visible dorsal and ventral rumen sacs and the entire rumen imaged ultrasonographically from the 9th intercostal space to the caudal flank on the left in 45 cows (mean ± standard deviation).

The visible height of the dorsal sac of the rumen was largest in the caudal flank (40.3 ± 6.33 cm; Figure 4), decreased cranially because of superimposition of the lung and measured 27.9 ± 8.74 cm at the 9th intercostal space (Difference to the caudal flank P < 0.05). In the left flank, the dorsal sac of the rumen was adjacent to the abdominal wall (distance 1.1 ± 1.38 cm). Cranially the distance between the abdominal wall and dorsal sac of the rumen increased gradually; it measured 10.3 ± 1.66 cm at the 9th intercostal space because of interposition of the spleen, which increases in thickness cranially. The mean thickness of the wall of the dorsal sac of the rumen was 0.3 cm.

### Left longitudinal ruminal groove

**Figure 5:**
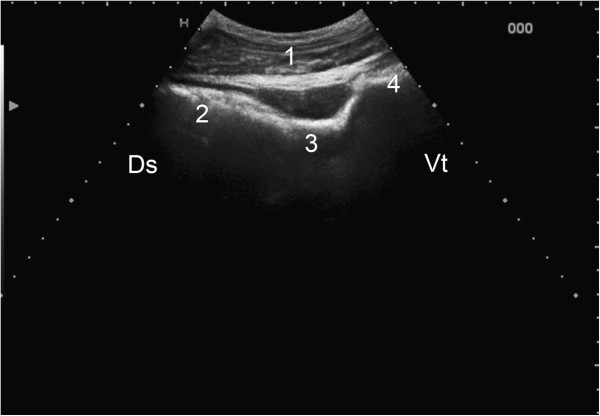
**Ultrasonogram of the longitudinal groove. **Ultrasonogram of the rumen of a three-year-old Holstein Friesian cow viewed at the level of the longitudinal groove at the 12th intercostal space. 1 Abdominal wall, 2 Dorsal sac of rumen, 3 Longitudinal groove, 4 Ventral sac of rumen, Ds Dorsal, Vt Ventral.

The left longitudinal ruminal groove separates the dorsal and ventral sacs of the rumen. It could be clearly identified at all examination sites because it appeared on ultrasonograms as a typical triangular notch (Figure 5), which was more distinct caudally than cranially.

### Ventral sac of the rumen from the left

The ventral sac of the rumen was visible in all cows from the 11th intercostal space to the caudal flank as well as at the 10th intercostal space in 22 cows (49%). The left longitudinal groove formed the dorsal border of the ventral sac of the rumen. In 21 cows, the ventral sac of the rumen extended across the ventral midline into the right hemiabdomen. The distance between the dorsal midline and the ventral margin of the ventral sac of the rumen varied little (Figure 3, Table 1). At all examination sites, the visible height of the ventral sac of the rumen was greater than the visible height of the dorsal sac (Figure 4); it was largest at the 12th intercostal space (62.6 ± 9.53 cm) and smallest at the 10th intercostal space (46.0 ± 8.65 cm; Table 1) (Difference to ICS 12 P > 0.05). The mean distance between the wall of the ventral sac and the abdominal wall varied from 4.6 cm to 6.1 cm. Other structures such as the abomasum, small intestines and omentum were occasionally seen ventrally between the abdominal wall and rumen. The mean thickness of the wall of the ventral sac of the rumen was 0.3 cm.

### Stratification of ruminal ingesta

The dorsal gas cap was imaged in all cows from the 12th intercostal space to the caudal flank. It was easily identified by characteristic reverberation artifacts, which disappeared abruptly at the transition to the fibre mat (Figure 2). It measured 20.5 ± 7.03 and 19.2 ± 6.33 cm at the 12th intercostal space and cranial flank, respectively, and 15.6 ± 5.35 cm at the caudal flank (Difference P > 0.05). The size of the dorsal gas cap changed during ruminal contractions. It was not possible to differentiate a fibre mat and a ventral fluid layer because the ingesta below the gas cap had a uniform ultrasonographic appearance.

### Rumen scanned from the right

The rumen was imaged from the right side in 21 cows (47%). It was visible at the 11th and 12th intercostal spaces and the cranial and caudal flank in one, five, 16 and 18 cows, respectively. The rumen was never imaged adjacent to the right abdominal wall because it was displaced medially by the liver, omasum and small and large intestines. The distance from the abdominal wall to the rumen at the 11th and 12th intercostal spaces and the cranial and caudal flank was 19.6 cm, 14.6 ± 1.79 cm, 11.7 ± 4.85 cm and 10.5 ± 3.65 cm, respectively. The ventral and dorsal sacs of the rumen could not be differentiated.

## Discussion

Assessing ruminal features such as wall thickness, size and stratification of ingesta is not possible by clinical examination alone. Our study has shown that at least some measure of objectivity in the assessment is achieved by using ultrasonography. Ultrasound examination yields exact information regarding echogenicity and thickness of the wall, the size of the dorsal gas cap and the ruminal height at different intercostal spaces and the flank; however, the fibre mat and fluid layer cannot be differentiated. Ultrasound examination yields exact information regarding echogenicity and thickness of the wall, the size of the dorsal gas cap and the ruminal height at different intercostal spaces and the flank. Based on previous reports [9,10] and the present study, the dorsal sac, the left longitudinal groove and the ventral sac of the rumen can always be imaged from the left abdominal wall in cows. This is also true for goats [12] and largely for calves as well [8], in which the rumen is visible ultrasonographically on the first day of life [14,15]. As reported in goats, the rumen wall appeared hyperechoic, which can be attributed to the tela submucosa and tela mucosa [16]; however, in agreement with other studies [9,10], the individual layers of the rumen wall could not be differentiated ultrasonographically.

Both the visible dorsal margin of the rumen and the left longitudinal groove ran from cranioventral to caudodorsal, whereas the ventral visible margin had a largely horizontal course. The course of the dorsal visible margin was due to superimposition of the lung. Dorsal and ventral ruminal margins were similar in nursing calves [8] and goats [11] except that in calves the rumen was sometimes imaged at the 7th and 8th intercostal spaces but not usually at the caudal flank. In goats, the rumen could also be imaged at the 8th intercostal space and always at the caudal flank. Visibility of the rumen at the caudal flank reflects the enormous growth of the rumen after the transition from milk to a roughage diet.

The rumen was largest at the 12th intercostal space and the cranial flank, which was in agreement with findings in nursing calves and goats. However, in contrast to goats, in which the dorsal sac of the rumen is larger than the ventral sac at all examination sites [11], the ventral sac of the rumen was always larger than the dorsal sac in the cows of this study. The size of the ventral sac changed little from cranial to caudal, whereas the apparent size of the dorsal sac, because of superimposition of the lung, increased from 46.0 cm in the 10th intercostal space to 62.6 cm in the 12th intercostal space.

Previous studies of the ultrasonographic visibility of the stratification of ruminal ingesta have yielded ambiguous results. There is agreement that in mature cattle [9,10], calves [8] and goats [11], the identification of the dorsal gas cap is straightforward because of typical reverberation artifacts, which are caused by the gas and run parallel to the rumen wall. Likewise, the transition from the gas cap to the fibre mat is easily recognised because of the abrupt cessation of these artifacts at the level of the ingesta. The differentiation of the transition from the fibre mat to the ventral fluid layer is more difficult. In a small study, the fibre mat could be distinguished as an echoic mass with gaseous inclusions and the fluid phase as a hypoechoic layer in two of three cows [9]. The findings of the present study are in agreement with those obtained from ten Jersey cross cows, in which differentiation of the fibre mat and fluid phase was also not feasible [10]. Considering the difficulties encountered in a relatively large number of cows investigated, we conclude that reliable ultrasonographic differentiation of fibre mat and ventral fluid phase in cows is the exception. This is in contrast to computed tomography, which allowed unequivocal differentiation of the two layers in calves [6] and goats [5]. However, despite this limitation, ultrasonographic findings of the rumen content supplement clinical findings; tympany caused by eructation problems can be quantified and possibly liquefaction of rumen contents, which is characteristic of grain overload, can be detected.

## Conclusions

This study has shown that ultrasonography is well suited for the detailed examination of the rumen of cows including position and size, wall thickness and size of the gas cap. The reference values obtained from a large number of dairy cows of different breeds add to the diagnostic tools that are available for the assessment of bovine patients. Ultrasonography produces measurements that can facilitate the assessment of the severity of diseases associated with ruminal enlargement such as reticulo-omasal stenosis, diseases associated with an enlarged dorsal gas cap such as tympany or those associated with liquefaction of the rumen content such as grain overload.

## Competing interests

The authors declare that they have no competing interests.

## Authors’ contributions

UB initiated and planned the study, he wrote the manuscript and made the figures. AS and LT carried out the ultrasonographic examinations under supervision of UB. All authors have read and approved the manuscript.

## Fundings

This study was financed by the University of Zurich, Switzerland.
